# Mammographic density, blood telomere length and lipid peroxidation

**DOI:** 10.1038/s41598-017-06036-y

**Published:** 2017-07-19

**Authors:** Natalie J. Erdmann, Lea A. Harrington, Lisa J. Martin

**Affiliations:** 1 Princess Margaret Cancer Centre, University Health Network, Toronto, ON M5G 2M9 Canada; 2 Institute for Research in Immunology & Cancer, Départment de Médécine, Université de Montréal, Montréal, QC H3T 1J4 Canada; 3 School of Biological Sciences, College of Science and Engineering, University of Edinburgh, The Kings Buildings, Mayfield Road, Edinburgh, UK

## Abstract

Extensive mammographic density is a strong risk factor for breast cancer, but may also be an indicator of biological age. In this study we examined whether mammographic density is related to blood telomere length, a potential marker of susceptibility to age-related disease. We measured mammographic density by a computer assisted method and blood telomere length using a validated PCR method. Urinary malondialdehyde (MDA), a marker of lipid peroxidation, was measured in 24 hour urine collections. In the 342 women examined telomere length was negatively correlated with age, was lower in postmenopausal compared to premenopausal women and in smokers compared to non-smokers, and was positively correlated with urinary MDA. Telomere length was not associated with percent mammographic density or dense area, before or after adjustment for risk factors and MDA. However, there was a significant interaction between telomere length and MDA in their association with mammographic density. At lower levels of MDA, mammographic density and telomere length were inversely associated; while at high levels of MDA, there was evidence of a J-shaped association between mammographic density and telomere length. Further work is need to replicate these results and to examine the association of mammographic density with age-related chronic disease and mortality.

## Introduction

Mammographic density (MD), which refers to variations in the radiological appearance of the breast, is one of the strongest known risk factors for breast cancer^1^. Dense breast tissue contains mainly fibroglandular tissue (epithelial cells and stroma)^2, 3^ and appears white on mammogram, whereas fatty tissue is radiolucent and appears dark. The amount of MD is highly heritable^4^ and large differences in MD are observed between women early in life^5^. The biological mechanism(s) for the increased breast cancer risk associated with MD is not yet known; however, MD may reflect the cumulative exposure of breast tissue to hormones and growth factors that stimulate cell proliferation and to DNA damage due to oxidative stress^1^. Further knowledge about the phenotype of extensive MD may improve our understanding of its cause and ultimately lead to effective interventions to prevent breast cancer.

While extensive MD is strongly associated with breast cancer risk, it may also be a marker of biological age. MD is greatest at young ages, declines with age, and is reduced by menopause (1). Higher MD is associated with reduced/delayed involution of breast tissue that occurs with age and menopause^6, 7^, and menopause occurred at a later age in a cohort of women with extensive MD compared to that expected in the general population^8^. Later menopause is associated with a higher risk of breast cancer, but also with lower risk of mortality^9, 10^. One study reported an association of higher MD with lower mortality in women without breast cancer^11^. To explore the possibility that MD may reflect a reduced susceptibility to age-related disease, we examined whether MD was associated with blood telomere length (TL), a potential marker of susceptibility to chronic disease^12^.

Telomeres, which consist of a variable number of repeat sequences that cap the ends of chromosomes to protect them from damage, shorten with cell division in most somatic cells^13^. Critically short telomeres can result in the onset of cellular senescence or apoptosis^14^. The length of telomeres in while blood cells at a given age varies considerably between individuals^15^. Shorter mean blood TL has been associated with higher risk of mortality^16–19^, coronary artery disease^20, 21^, Alzheimer’s disease^22, 23^, diabetes^24^, and several solid cancers^25, 26^.

Although blood TL shortens with age during adult life^27, 28^ and may be inversely associated with markers of oxidative stress^29, 30^, TL is highly heritable and differences in blood TL in adults are likely largely determined at birth and as a result of changes during early growth in childhood^31^. Due to its high heritability, TL in blood also reflects the length of telomeres in other tissues, and longer telomeres may reflect greater replicative potential and ability for tissue repair which influences susceptibility to age related diseases and longevity^31^.

The primary goal of this study was to examine the association between MD and blood TL. If extensive MD is a marker of reduced susceptibility to age-related chronic disease, we would expect to see that it is positively associated with blood TL. Since both MD and TL have been reported to be associated with oxidative stress^29, 30, 32–34^; our secondary goal was to determine whether the relationship between MD and blood TL is influenced by levels of urinary MDA excretion, a marker of lipid peroxidation (oxidative stress). We first validated the PCR method^35^ for measuring TL against the Southern blotting method (Telomere Restriction Fragment analysis; TRF), and then measured blood TL in a sample of women with a wide range of MD.

## Methods

### General method

We selected pre- and post-menopausal women without breast cancer but with different degrees of mammographic density, collected information about risk factors, and obtained biological samples (serum, urine and DNA) under standardized conditions^36^. Blood TL was measured using a quantitative PCR method^35^ that was validated in our laboratory. All procedures were carried out in accordance with the Tri-Council Policy Ethical Conduct for Research Involving Humans (2005). Ethical approval for the study protocol was obtained from the Human Subjects Review Committee at the University of Toronto and the University Health Network Research Ethics Board. All subjects provided signed informed consent.

### Selection of subjects and recruitment

Between 1994 and 1997 potentially eligible women were identified from mammographic units in Toronto, Ontario, Canada. Approximately equal numbers of pre- and post-menopausal women were selected in from each of five percent density categories: <10%, 10–25%, 25–50%, 50–75%, and >75%. MD was subsequently classified by quantitative methods that are described below.

Premenopausal women were eligible if they were menstruating regularly, not pregnant or breast-feeding, and had not had a hysterectomy or oophorectomy. Postmenopausal women were eligible if they had spontaneous amenorrhea for at least 12 months, or had had a hysterectomy and were 50 years of age or older, or had had a bilateral oophorectomy at any age. Women taking any type of exogenous hormone preparation, who had had breast augmentation or reduction, or had previously been diagnosed with breast cancer were excluded. In total, 382 women agreed to participate in the study, representing 88% of those who were contacted and found to be eligible.

### Epidemiological Risk Factors and Anthropometric Measures

Information about epidemiologic risk factors for MD and breast cancer was collected by an interviewer-administered questionnaire. Subjects were weighed on a balance scale and measured for height.

### Measurement of MD

Mammograms of the craniocaudal view of one breast for each subject (side randomly selected) were digitized using a Lumisys model 85. Total breast area and dense area were measured on randomly ordered images using a computer assisted method^37^ by one reader who was blinded to subject identity and characteristics. Percent MD was calculated by dividing dense area by total area and multiplying by 100. Non dense area (fat) was calculated by subtracting dense area from the total area of the breast. Inter- and intra-batch reliability of mammographic measures was at least 0.9.

### DNA and Urine Collection

For premenopausal women, blood and 24 hour urine samples were collected between days 20–24 of the menstrual cycle (luteal phase). Buffy coat was separated and stored at −70 °C until DNA extraction. Precipitated genomic DNA was treated with RNase A and RNase T1 to remove RNA contamination and isolated DNA was hydrolyzed using nuclease P1 and alkaline phosphatase. Extracted DNA was stored at −80 °C until analysis of TL.

During collection urine was stored at room temperature in containers with 5 ml of 5N hydrochloric acid and was couriered to the laboratory the morning after completion of collection. Total urine volume was measured and aliquots were frozen at −70 °C until analysis.

### Blood telomere length measurement

To validate the quantitative PCR method, TL was measured in genomic DNA using the standard Southern blotting TRF method and PCR. DNA was extracted from whole blood obtained from 100 subjects who were randomly selected from participants in other ongoing studies^5, 38^ stratified by age to ensure a wide age range (15–76 years).

### Terminal Restriction Fragment (Southern blotting)

Two μg of DNA was digested with HinfI and RsaI, resolved on a 0.5% w/v agarose gel at low voltage, transferred to a nylon membrane and probed with radiolabelled 5′-(CCCTAA)_3_-3′ telomeric probe. The membrane was reprobed with radiolabelled 1 Kb Plus DNA Ladder (Invitrogen) and the scanned images were merged. Mean telomere length restriction fragment (TRF) values were calculated using Image J and Excel software from at least 3 independent experiments using the method described by Chai *et al*.^39^. The coefficient of variation (CV) for TRF triplicates was 1.6%.

### Quantitative PCR

Quantitative PCR was based on a method described by Cawthon^35^, where average telomere length (the T/S ratio) is determined as the factor by which the experimental sample differs from a reference DNA sample in its ratio of telomere repeat copy number to a single gene copy number using SYBR Green as a detector. A serially diluted reference DNA sample (5 to 60 ng per well) was used to generate a standard curve and included a “no template control” (i.e. primers and other components but no genomic DNA) in all PCR reactions. Telomeric PCRs and single gene PCRs were performed in separate 384-well plates, in triplicate, using Power SYBR Green PCR Master Mix (Applied Biosystems) and 20 ng DNA per well (total volume 10 μl). Telomeric C_t_ (“T”) was measured using primers from Epel *et al*.^30^ at a final concentration of 300 nM, and single gene C_t_ (“S”) was measured with primers for 36B4 (acidic ribosomal phosphoprotein PO) from Cawthon *et al*.^35^ at the recommended concentrations. Reactions were performed in a Prism 7900HT thermocycler (Applied Biosystems) using the following thermal cycling profiles: telomeric amplification 95 °C 10 min, followed by 22 cycles of 95 °C 15 s, 56 °C 30 s and 72 °C 30 s; 36B4 amplification 95 °C 10 min, followed by 30 cycles of 95 °C 15 s and 58 °C 1 min. The T/S value for each sample was calculated using the formula T/S = 2^Ct 36B4-Ct tel^°^mere^, where C_t_ values were obtained from at least 3 triplicate measurements on each of 3 plates (n ≥ 9), and accepted with standard deviations of <0.2. Each experimental sample T/S ratio was then divided by the reference sample T/S ratio to determine the final relative telomere length (RTL).

### Validation of PCR

We used samples from 25 of the 100 subjects to initially optimize the procedures for PCR. In these samples, the correlation between TL measured by PCR and TRF was 0.84 and reliability was 0.80 based on repeated measurements. We carried out the formal validation using DNA from the remaining 75 subjects (analyzed in 2 batches of size 35 and 40 subjects respectively that were analyzed about 6 months apart) with a wide age range (15.3–76.4 years; mean = 42.5, SD18.7). Mean TRF was 6.33 kbp (SD 0.40, range 5.32 to 7.32) and mean RTL was 1.03 (SD 0.18; range 0.63 to 1.59). There was one potential outlier for the PCR measurements (1.59) but its exclusion had virtually no effect upon the results and it was retained in the dataset. The two measures of blood TL were highly correlated (r = 0.73, 95% CI: 0.61, 0.82; see Supplementary Fig. S1). After adjustment for batch, the correlation between measures was 0.83 (95% CI: 0.74, 0.89). Both measures were strongly inversely correlated with age (r = −0.71, 95% CI: −0.81, −0.58 for TRF and r = −0.62, 95% CI: −0.74, −0.46 for RTL; see Supplementary Fig. S2).

### Measurement of RTL for Mammographic Density Study

A total of 351 DNA samples were thawed at 4 °C, and DNA concentrations were re-measured using a NanoDrop Spectrophotometer (Thermo Fisher Scientific). Quantitative PCR was performed using 10 ng DNA per well and RTL was calculated as described above. The samples were divided into 4 batches (plates). Each batch contained 10 intra-batch repeats and 20 DNA samples were measured in each batch (inter-batch repeats). In addition, repeat samples of DNA from 35 randomly selected subjects were distributed randomly throughout the batches to measure reliability. The intra-class correlation for the intra-batch and inter-batch repeats was 0.9. The repeat measures for 35 samples randomly dispersed across batches were highly correlated at 0.80, and after exclusion of one outlier, the correlation was 0.87. The inter-batch CV for RTL measured by PCR was 4.6%.

### MDA Measurement

MDA in urine was measured in triplicate by HPLC determination of thiobarbituric acid derivatives as described by Bird *et al*.^40^. Samples were extracted with trichloracetic acid and then heated with thiobarbituric acid. The thiobarbituric acid-MDA complex was separated using HPLC and the absorbance measured at 535-nm.

### Statistical Methods

The distributions of non dense breast area and MDA measurements were highly skewed and both variables were expressed as the natural log to improve normality. Blood telomere measurements (TRF and PCR) were approximately normally distributed or symmetrical and were not transformed. To improve distribution of residuals plotted against the predicted values from the multiple linear regression analyses (see below), percent MD and dense area were square root transformed and waist circumference was expressed as the negative inverse.

The characteristics of premenopausal and postmenopausal women were compared using 2 sample t-tests for symmetrically distributed continuous variables, Wilcoxon rank sum tests for continuous variables whose distributions were skewed, and Chi square test for categorical variables. The univariate association between RTL and mammographic measures was assessed by Spearman correlation and simple linear regression. We first examined the univariate associations of age, anthropometrics, breast cancer risk factors and MDA with RTL and mammographic measures using simple linear regression and then determined their independent associations with the outcomes using multiple linear regression.

Multiple linear regression was also used to assess the relationship between RTL and mammographic measures (outcome measure) after adjustment for potential confounders and to test selected multiplicative interaction terms with RTL (urinary MDA, menopausal status and waist circumference). Linearity of the association of RTL with mammographic measures was assessed by examining RTL as a categorical (tertiles) variable.

Data analyses were carried out using the SAS statistical software package (version 9.3 SAS Institute Inc., Cary, NC, USA). All tests were 2-sided and results were considered statistically significant at p < 0.05.

### Data Availability

Access to data generated and analysed for this study requires approval from the University Health Network Research Ethics Board for Oncology, and adherence to the guidelines for the protection of privacy of research subjects laid down by the Canadian Institute for Health Research. Requests for the data may be sent to the corresponding author.

## Results

### Study Population

DNA was available for 92% (n = 351) of the 382 women in the original MD study^36^. RTL results for 8 subjects were excluded as their replicate measures had a SD greater than 0.2, and one outlier (RTL of 2.8) was excluded leaving 342 subjects for analysis.

Table 1 shows selected characteristics of the subjects presented for the whole group and divided by menopausal status. In the whole group, the mean age was 50.4 (SD 7.2), body mass index (BMI) was 25.6 (SD 5.9), and percent MD was 40.2% (SD 25.3). Because women were selected to represent a wide range of percent MD, and percent MD is strongly associated with weight (33), the sampling procedure also resulted in a wide range in weight (38.6 to 152.9 kg). Compared to premenopausal women, postmenopausal women were older and weighed more, had lower percent MD, and RTL, and higher non dense area and urinary MDA.

**Table 1:** Selected characteristics of subjects.

	All Women (n = 342)	Premenopausal (n = 173)	Postmenopausal (n = 169)	p value^a^
Age (years)	50.4 (7.2)	44.8 (4.7)	56.0 (4.4)	<0.0001
Weight (kg)	68.5 (16.4)	66.9 (15.6)	70.1 (17.0)	0.07
BMI (kg/m^2^)	25.6 (5.9)	25.1 (5.7)	26.0 (6.1)	0.12
Waist (cm)	73.5 (13.9)	71.6 (13.2)	75.4 (14.3)	0.01
Parous (%)	72.5	70.5	74.6	0.40
Family History of breast cancer (%)^b^	22.5	23.7	21.3	0.60
Current Smoking (%)	12.6	14.5	10.7	0.29
Percent Mammographic Density (%)	40.2 (25.3)	43.7 (25.7)	36.6 (24.5)	0.01
Dense Area (cm^2^)	46.2 (32.6)	48.7 (31.4)	43.5 (33.6)	0.14
Non Dense Area (cm^2^- log)	4.19 (0.83)	4.08 (0.84)	4.30 (0.82)	0.02
Relative Telomere Length	1.03 (0.20)	1.10 (0.21)	0.97 (0.18)	<0.0001
Urinary MDA (umol/l – log)	7.96 (0.48)	7.85 (0.51)	8.07 (0.43)	<0.0001

Continuous variables are presented as mean (SD) and categorical variables as percentage.

^a^p value for comparison of pre and postmenopausal women. Two-sided two-sample t-test for continuous variables and Chi square test for categorical variables.

^b^Percentage of subjects with at least one first degree family member with breast cancer.

### Univariate association of mammographic measures and RTL

**Figure 1:**
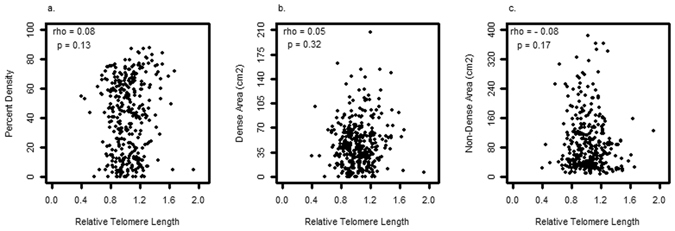
Correlation between relative telomere length and percent mammographic density (**a**), dense area (**b**), and non dense area (**c**). Rho = Spearman correlation coefficient.

There were no significant correlations between mammographic measures and RTL in univariate analysis (Fig. 1). In premenopausal women, the correlation of percent MD, dense area and non dense area with RTL was 0.05, 0.06, and −0.09 respectively, and in postmenopausal women were 0.01, 0.04, and 0.01 respectively (all p-values ≥ 0.3).

### Association of covariates with mammographic density, RTL and MDA

Prior to examining the influence of risk factors and MDA on the association of RTL with mammographic measures, we examined the associations of the risk factors with mammographic measures, RTL and MDA using simple linear regression (Supplementary Tables S1 and S2) and multiple regression (Table 2 and Supplementary Table S3). Multiple regression was performed in the subset of subjects with urinary MDA measures (n = 305), but the results for variables other than MDA were similar in the larger set of 342 women. Among the measures of body size examined (weight, body mass index and waist circumference), waist circumference was the most strongly associated with the mammographic measures, and was the measure of body size used in all multivariable analyses.

**Table 2:** Factors associated with percent mammographic density, relative telomere length and urinary MDA (multiple regression; n = 305).

Characteristic	Percent Mammographic Density (square root)^a^			Relative telomere length^a,b^			Urinary MDA (log)^a^		
	Beta	SE	p value	Beta	SE	p value	Beta	SE	p value
Age (years)	−0.003	0.02	0.90	−0.005	0.002	0.02	−0.008	0.006	0.16
Waist Circumference^c^ (cm)	−679.6	45.0	<0.0001	−6.88	4.62	0.14	16.82	11.5	0.15
Height (cm)	−0.003	0.02	0.85	0.002	0.002	0.30	0.01	0.004	0.004
Age at Menarche (years)	0.04	0.07	0.52	−0.009	0.007	0.21	−0.02	0.02	0.26
Parity (Yes vs no)	−0.20	0.23	0.23	−0.005	0.02	0.83	−0.02	0.06	0.78
Age at First Child (years)	0.06	0.02	0.01	0.002	0.002	0.47	0.007	0.006	0.22
Menopausal Status Post vs Premenopausal	−0.05	0.34	0.88	−0.08	0.05	0.02	0.32	0.09	0.0003
Family History of Breast Cancer Yes vs No	0.10	0.24	0.70	−0.02	0.03	0.43	0.02	0.06	0.77
Current Smoking Smoker vs non smoker	−0.42	0.32	0.19	−0.07	0.03	0.03	0.15	0.08	0.07
Urinary MDA (log)	0.37	0.23	0.10	0.05	0.02	0.02	n/a	n/a	n/a
R square for model	49%			23%			11%		

MDA = malondialdehyde; RTL = relative telomere length; SE = standard error; n/a = not applicable.

^a^Outcome measure for regression analysis.

^b^Adjusted for batch of PCR analysis; ^c^Negative inverse transformed.

In multivariable models (Table 2), waist circumference was strongly inversely associated with percent MD (p < 0.0001), while later age at first birth was associated with higher percent density (p = 0.01). The positive association of urinary MDA with percent MD was not statistically significant (p = 0.10). The associations with dense area were similar to those observed for percent MD; whereas for non dense area they were in the opposite direction, but only the positive association with waist circumference was statistically significant (Supplementary Table S3).

Higher age was negatively associated with RTL (p = 0.02) and premenopausal women had longer RTL than postmenopausal women (p = 0.02) (Table 2). Non-smokers had longer RTL than smokers (p = 0.03) and urinary MDA was positively associated with RTL (p = 0.02). Greater height (p = 0.004), smoking (p = 0.07), and being postmenopausal versus premenopausal (p = 0.0003) were associated with higher levels of MDA.

There were no associations of family history of breast cancer, age at menarche or parity with any of the outcome measures in univariate or adjusted analyses.

### Association of mammographic measures and RTL after adjustment for covariates

Table 3 shows that RTL was not significantly associated with any of the mammographic measures before or after adjustment for the risk factors (from Table 2) and MDA. When we categorized RTL into tertiles, the adjusted (least square) means for percent MD (square root) were 5.98, 5.72, 5.75 (ANOVA p = 0.57; p for trend = 0.40).

**Table 3:** Association of relative telomere length with percent mammographic density and dense area before and after adjustment for risk factors and urinary MDA (n = 305).

Variable	Percent Mammographic Density (square root)^a^			Dense Area (square root)^a^			Non dense area (log)^a^		
	Beta	SE	p value	Beta	SE	p value	Beta	SE	p value
RTL alone^b^	0.82	0.71	0.25	0.82	0.73	0.26	−0.41	0.25	0.10
RTL adjusted for risk factors^c^	−0.32	0.57	0.58	0.21	0.75	0.78	0.05	0.16	0.77
RTL adjusted for Risk Factors and MDA^d^	−0.44	0.57	0.44	0.05	0.75	0.95	0.07	0.17	0.66
Interaction between RTL and MDA^e^	3.54	1.06	0.001	2.82	1.41	0.05	−1.17	0.31	0.0002

RTL = relative telomere length; MDA = malondialdehyde; SE = standard error.

^a^Outcome measure for regression analysis.

^b^Adjusted for PCR batch only.

^c^Adjusted for PCR batch and risk factors (from Table 2), except for MDA.

^d^Adjusted for PCR batch, risk factors and MDA.

^e^Interaction term for RTL*MDA, adjusted for main effects and risk factors.

**Figure 2:**
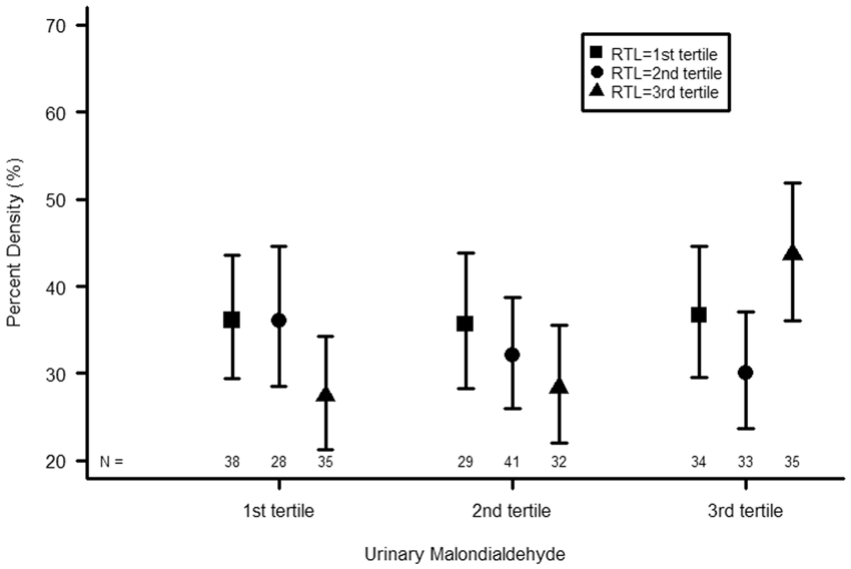
Percent mammographic density by tertiles of urinary MDA and RTL. Results are shown as least square means and standard error bars. P value for interaction between tertiles of MDA and tertiles of percent density = 0.02. MDA = malondialdehyde; RTL = relative telomere length.

There were no significant interactions of RTL with menopausal status or waist circumference for any of the mammographic measures (p > 0.56 for all). However, there was a significant interaction between RTL and urinary MDA (Table 3, last row; p = 0.001 for percent MD; p = 0.05 for dense area and p = 0.0002 for non dense area). To illustrate this interaction for percent MD, we categorized RTL and MDA into tertiles and show the least square means of percent MD (back transformed) for each category in Fig. 2. In the lower 2 tertiles of MDA, longer RTL was associated with lower percent MD. However, in the highest tertile of MDA, these results suggest a J-shaped curve, with highest percent MD in the highest tertile of RTL. The interaction is illustrated in the same way for dense area and non dense area in Supplementary Fig. S3.

Because waist circumference was strongly correlated with percent MD and also influenced the association of MDA with mammographic measures, we examined the interaction term in the model for percent MD without adjustment for any other covariates as a sensitivity analysis. The interaction between RTL and MDA was highly significant (Beta = 3.93, p = 0.007).

## Discussion

To explore the possibility that MD, a strong risk factor for breast cancer, may be related longevity or lower risk of age-related chronic disease, we examined whether MD was associated with blood TL, a potential marker of susceptibility to age-related chronic disease. The overall results of our study show no association between MD and TL and are in agreement with the one other study on this relationship^41^. However, we also examined the influence of lipid peroxidation on the association of MD and TL, and we found that in women with low/moderate levels of urinary MDA excretion, there was an inverse association between MD and TL; whereas in women with high levels of urinary MDA excretion, women with the highest relative blood TL had the highest MD.

The association of blood TL with breast cancer risk has been inconsistent^25, 26^. Retrospective case-control studies have reported no significant difference in blood TL between breast cancer cases and controls^42–45^, and higher breast cancer risk associated with shorter telomeres^46^ and longer telomeres^47–49^. Of the four prospective studies published to date, one reported no association of TL with breast cancer risk^50^ and three reported that shorter TL was associated with higher breast cancer risk^46, 51, 52^; however, only the results of Qu *et al*.^52^ were statistically significant. Our observation of an inverse association of TL with MD in women with low/moderate levels of urinary MDA excretion are consistent with these results. Qu *et al*.^52^ also reported a reverse J-shaped association; with shorter and longer telomeres being associated with higher breast cancer risk. In women with high urinary MDA excretion, we observed that women in the highest tertile of blood TL had the highest percent MD. Together these results do not rule out the possibility that, in some contexts, longer TL could be associated with higher MD and higher breast cancer risk.

We know of only one study that examined markers of oxidative stress and blood TL with breast cancer risk^44^. Shen *et al*. reported that urinary excretion of isoprostane and 8-oxo-7,8-dihydrodeoxyguanosine (markers of lipid peroxidation and DNA damage from oxidative stress respectively) were not associated with breast cancer risk, and did not modify the association of TL with breast cancer risk.

In agreement with other studies^41, 51, 52^, we did not observe any association of blood TL with traditional breast cancer risk factors, specifically age at menarche, parity, age at first child or family history of breast cancer. Factors associated with oxidative stress such as smoking, higher body weight, psychological stress, insulin resistance and C-reactive protein levels (a marker of inflammation) are associated with shorter blood TL^15, 18, 30, 53, 54^. Urinary isoprostane excretion, a marker of lipid peroxidation, has been reported to be inversely associated with blood TL in some^29, 30^, but not all^44^ studies. Consistent with these observations, we found that higher waist circumference, postmenopausal status, and smoking were associated with shorter TL and higher urinary MDA excretion, also a marker of lipid peroxidation. Unexpectedly, we found that urinary MDA excretion was positively associated with blood TL. The reason for this positive association unclear. We^55^ and others^56^ have reported little correlation between measures of MDA and isoprostane, and that isoprostane levels were more strongly positively correlated with body mass index compared to MDA levels. Therefore, urinary MDA and isoprostane excretion may reflect different cellular networks and/or different sensitivities to exposures. For example, diet is a potential source of MDA excreted in urine^57^ and MDA is as a by-product of thromboxane synthesis in platelets via the cyclo-oxygenase cascade^58^, while urinary isoprostane is not related to these sources.

The strengths of our study include the large sample size, wide range of MD, and use of validated, reliable measurements of MD^59, 60^ and TL. Few of the previous breast cancer studies validated their PCR-based TL measurements against the standard TRF method (Southern blotting), and several of them reported higher inter- and intra-assay CVs for the PCR-based TL measurements (14 to 28%)^42, 44, 51^ compared with the inter-assay CV of 4.6% reported by our lab and other laboratories with extensive experience using these methods^35, 61^.

A potential limitation of this study is that we measured mean TL in all white blood cells combined, and different blood cell types vary in the length of their telomeres^62, 63^. Mean TL is positively correlated with TL in all white blood cell types examined, but the relationship was strongest for CD8+ T cells^63^, which showed the strongest negative correlation of TL with age, and are the cells most reflective of immunosenescence^63^. It is possible that TL in particular cell types are associated with MD, or that differences in the distribution of cell types obscured a relationship between TL and MD.

In addition, the PCR method measures the mean TL over all chromosomes combined. The small percentage of critically short telomeres, rather than the mean TL, may be more important to cell viability and chromosomal stability^64–67^, and TL on particular chromosomes may be important for certain types of cancer^68^. For example, Zheng *et al*. showed that shorter telomere length in chromosome 9 was strongly associated with breast cancer risk while mean telomere length was not associated with breast cancer risk^68^.

In conclusion, our overall results show no association between MD and blood TL and are consistent with the one previous study on this association^41^. However, our study suggests that this relationship may depend on the level of oxidative stress. At low to moderate levels of urinary MDA, the association between MD and blood TL was inverse suggesting that higher MD may be associated with higher susceptibility to age-related chronic diseases. In contrast, there may be a subgroup of women (with high level of MDA excretion) who have high MD and long telomeres and therefore may be less susceptible to age-related chronic disease. Further work is needed to replicate these results and to further understand the relationships between TL, MD and markers of oxidative stress. In addition, the association of MD with outcomes other than breast cancer needs to be examined to provide insights into the etiology of MD and to aid in the development of safe and effective interventions to reduce breast cancer risk.

## Electronic supplementary material

Supplementary Information
