# Supplementary material for: Mammographic density, blood telomere length and lipid peroxidation
Source: Sci Rep. 2017 Jul 19;7:5803. doi: 10.1038/s41598-017-06036-y (PMC5517610; doi:10.1038/s41598-017-06036-y)
Supplement: Supplementary file 1 — Supplementary Information [file 41598_2017_6036_MOESM1_ESM.pdf]

Mammographic density, blood telomere length and lipid peroxidation

Erdmann, Natalie J, Harrington, Lea A, and Martin, Lisa J

Supplementary Information.

Supplementary Table S1. Univariate association of risk factors with percent density, relative telomere length and urinary MDA (simple linear regression)

	Percent Density (Square root) (n=342)		Relative Telomere Length (n=342)		Urinary MDA (log) (n=305)	
	B/Mean (SE)[a]	P value	B or mean (SE)	P value	B or mean (SE)	P value
Age (years)	-0.04 (0.02)	0.02	-0.008 (0.001)	<0.0001	0.008 (0.004)	0.04
BMI (kg/m^2)	-0.26 (0.02)	<0.0001	-0.003 (0.002)	0.07	0.006 (0.005)	0.20
Weight (kg)	-0.09 (0.01)	<0.0001	-0.0009 (0.0007)	0.16	0.004 (0.002)	0.03
Height (cm)	-0.03 (0.02)	0.09	0.001 (0.002)	0.39	0.013 (0.004)	0.002
Waist (cm)[b]	-692.3(39.6)	<0.0001	-6.8 (4.6)	0.14	26.5 (11.4)	0.02
Age at Menarche (years)	0.14 (0.08)	0.08	-0.002 (0.007)	0.74	-0.007 (0.02)	0.66
Parity (%)						
Yes	5.74 (0.15)		1.03 (0.01)		7.96 (0.03)	
No	6.19 (0.25)	0.13	1.04 (0.02)	0.62	7.96 (0.05)	0.95
Age at 1st child (years) [b]	0.10 (0.03)	0.0003	0.006 (0.002)	0.007	0.002 (0.006)	0.71
Menopausal Status						
Pre	6.16 (0.18)		1.10 (0.01)		7.85 (0.04)	
Post	5.55 (0.18)	0.02	0.97 (0.01)	<0.0001	8.07 (0.04)	<0.0001
Family History of Breast Cancer						
Yes	6.30 (0.27)		1.04 (0.02)		7.96 (0.06)	
No	5.74 (0.15)	0.07	1.03 (0.01)	0.95	7.96 (0.03)	0.95
Current Smoker						
Yes	5.48 (0.37)		0.99 (0.03)		8.05 (0.08)	
No	5.92 (0.14)	0.27	1.04 (0.01)	0.13	7.95 (0.03)	0.23
Telomere Length	0.79 (0.64)	0.22	--	--		
Urinary MDA[c]	-0.12 (0.29)	0.69	0.03 (0.02)	0.24	--	--

MDA = malondialdehyde

[a] expressed as beta for continuous variables and mean for categorical variables

[b] -1/waist; [b] n =243 for age at first child; [c] n=305

Supplementary Table S2. Univariate association of risk factors with dense area and non dense breast area (simple linear regression).

	Dense Area (cm^2)<br>(square root)<br>(n=342)		Non dense area (cm^2)<br>(log)<br>(n=342)	
	B or Mean (SE) [a]	**P value**	B or mean (SE) [a]	P value
Age (years)	-0.03 (0.02)	0.17	0.017 (0.006)	0.005
BMI (kg/m^2)	-0.14 (0.02)	<0.0001	0.10 (0.005)	<0.0001
Weight (kg)	-0.05 (0.01)	<0.0001	0.04 (0.002)	<0.0001
Height (cm)	-0.02 (0.02)	0.34	0.01 (0.006)	0.06
Waist (cm)[b]	-378.5 (53.4)	<0.0001	274.5 (11.5)	<0.0001
Age at Menarche (years)	0.06 (0.09)	0.51	-0.06 (0.03)	0.02
Parity (%)<br>Yes<br>No	 6.26 (0.16)<br>6.43 (0.26)	 <br>0.57	 4.24 (0.05)<br>4.05 (0.09)	 <br>0.06
Age at 1st child (years) [c]	0.10 (0.03)	0.0005	-0.02 (0.009)	0.007
Menopausal Status<br>Pre<br>Post	 <br>6.54 (0.19)<br>6.07 (0.19)	 <br><br>0.09	 <br>4.08 (0.06)<br>4.30 (0.06)	 <br><br>0.02
Family History of Breast Cancer<br>Yes<br>No	 <br>6.67 (0.29)<br>6.20 (0.16)	 <br><br>0.15	 <br>4.04 (0.09)<br>4.23 (0.05)	 <br><br>0.09
Current Smoker<br>Yes<br>No	 5.64 (0.38)<br>6.40 (0.15)	 <br>0.06	 4.16 (0.13)<br>4.19 (0.05)	 <br>0.83
Telomere Length	0.91 (0.67)	0.17	-0.33 (0.22)	0.14
Urinary MDA[d]	0.22 (0.30)	0.47	0.11 (0.10)	0.30

MDA = malondialdehyde

[a] expressed as beta for continuous variables and mean for categorical variables;

[b] -1/waist; [c] n =243 for age at first child n=305

Supplementary Table S3

Factors associated with dense and non dense area (multiple regression; n=305).

**Variable**	Dense Area (cm²) (square root) (n=342)		Non Dense Area (cm²) (log) (n=342)	
	Beta (SE)	p value	Beta (SE)	p value
Age (years)	0.01 (0.03)	0.62	0.01 (0.007)	0.06
Waist Circumference (cm)[a]	-342.0 (58.9)	<0.0001	278.9 (13.1)	<0.0001
Height (cm)	-0.006 (0.02)	0.78	0.0001 (0.005)	0.98
Age at Menarche (years)	0.05 (0.09)	0.58	-0.005 (0.02)	0.78
Parity (Yes vs no)	-0.10 (0.30)	0.73	0.07 (0.07)	0.32
Age at First Child (years)	0.08 (0.03)	0.01	-0.009 (0.006)	0.17
Menopausal Status (Pre vs post)	-0.22 (0.44)	0.62	-0.09 (0.10)	0.36
Family History of Breast Cancer (Yes vs No)	0.24 (0.32)	0.46	0.02 (0.07)	0.83
Current Smoking (Yes vs No)	-0.72 (0.42)	0.09	0.002 (0.09)	0.98
Urinary MDA[b] (log)	0.51 (0.30)	0.09	-0.08 (0.07)	0.23
R square for model	16%		64%	

MDA = malondialdehyde

[a] -1/waist

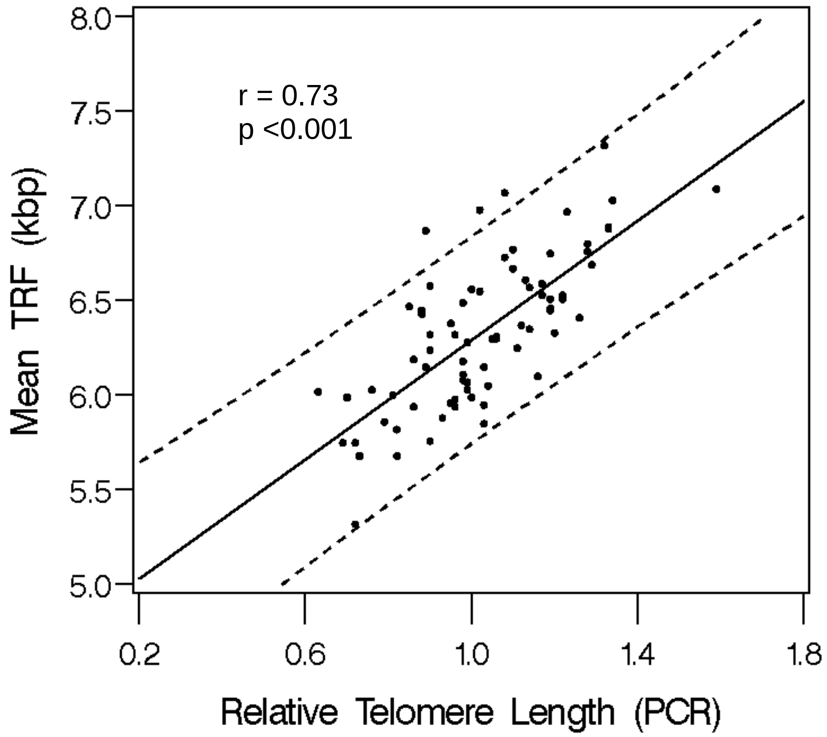

Supplementary Figure S1. Correlation between telomere length measured by the standard TRF method and PCR (relative telomere length) (n= 75 women). The correlation increased to 0.83 after adjustment for batch. Solid line shows the best fitting line from linear regression (TRF = 1.6 xT/S ratio + 4.7). Dotted lines show the 95% confidence intervals for the predicted values.

a

b

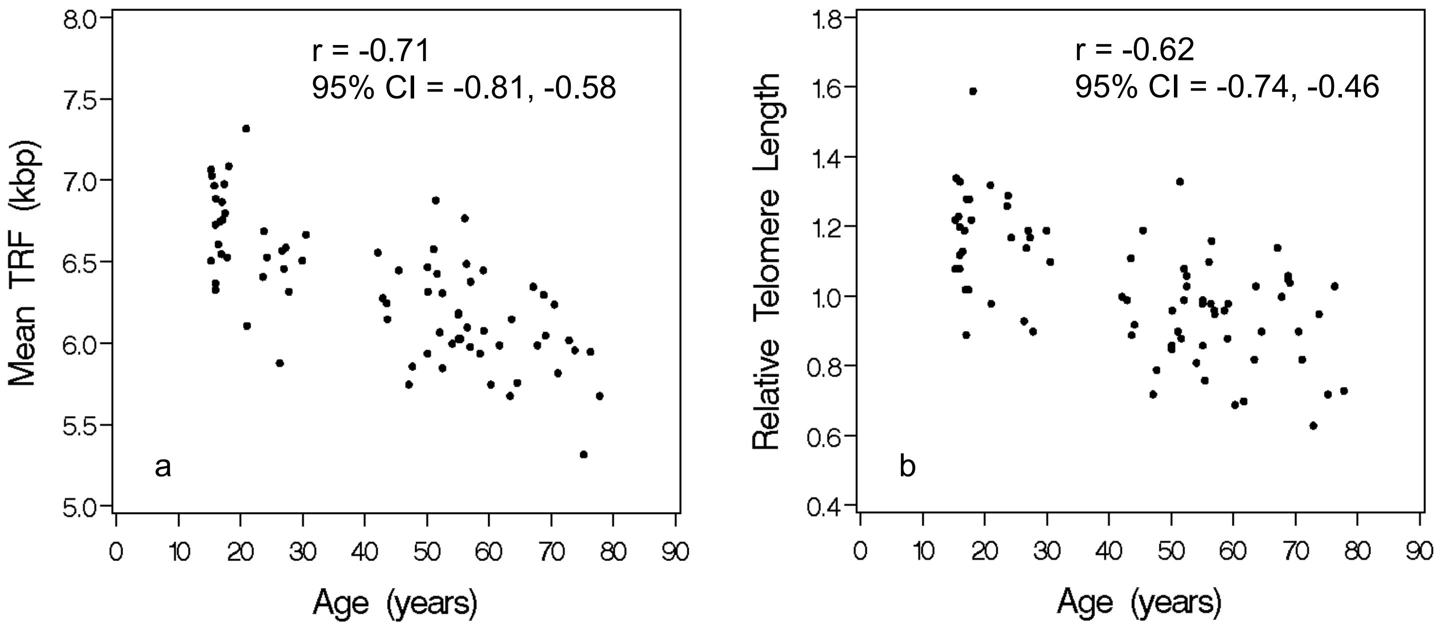

Supplementary Figure S2. Correlation of age with mean telomere length measured by TRF (a) and relative telomere length measured by Q-PCR (b) (n=75 women)

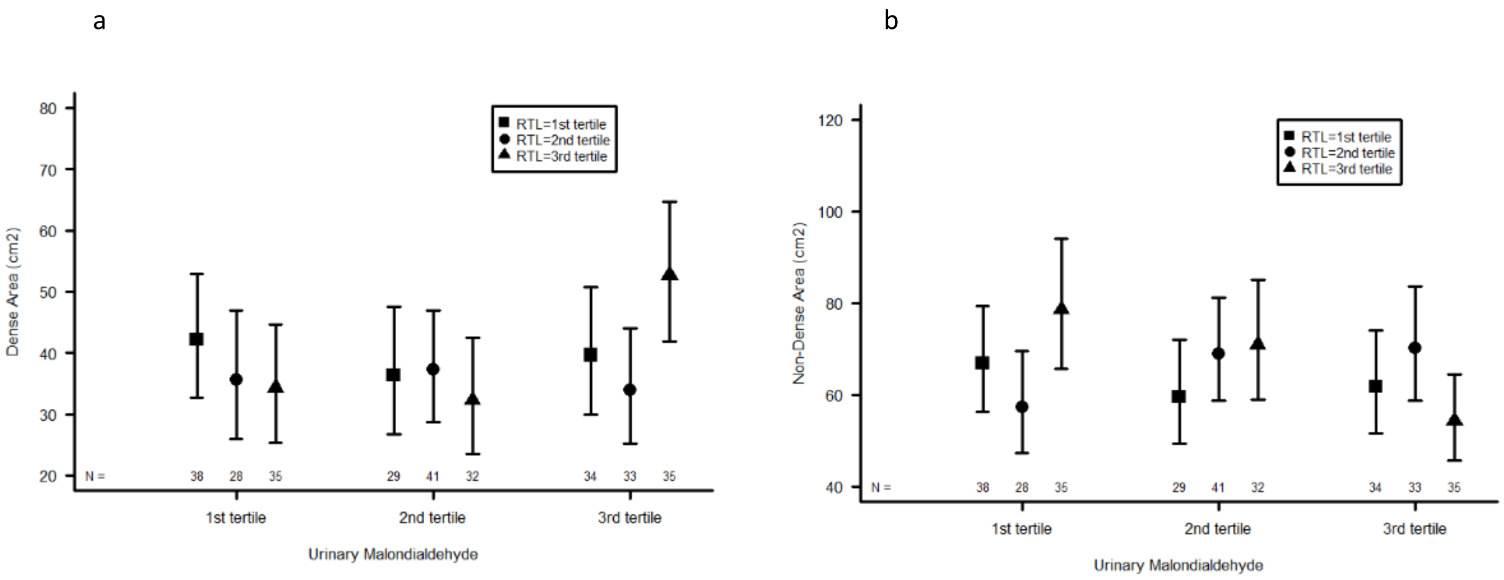

Supplementary Figure S3. Dense (a) and non dense area (b) by tertiles of urinary MDA and RTL. Results are shown as least square means and standard error bars.
